# Monitoring tert-Butylhydroquinone Content and Its Effect on a Biolubricant during Oxidation

**DOI:** 10.3390/molecules27248931

**Published:** 2022-12-15

**Authors:** Sergio Nogales-Delgado, Agustina Guiberteau Cabanillas, Ángela García Romero, José María Encinar Martín

**Affiliations:** 1 Department of Applied Physics, University of Extremadura, Avda. de Elvas s/n, 06006 Badajoz, Spain; 2 Department of Analytical Chemistry, University of Extremadura, Avda. de Elvas s/n, 06006 Badajoz, Spain; 3 Department of Chemical Engineering and Physical-Chemistry, University of Extremadura, Avda. de Elvas s/n, 06006 Badajoz, Spain

**Keywords:** TBHQ, frying oil, fatty acid methyl esters, pentaerythritol, transesterification, homogeneous catalysis, spectrophotometry, voltammetry, Rancimat method, viscosity, acid number

## Abstract

The use of biolubricants as a replacement for petroleum-based products is becoming more and more important, due to the current global energy and crude oil scenario. Thus, the production of biolubricants (which could take place in biorefineries) should be as efficient as possible, obtaining high-quality products with suitable viscosity or oxidation stability values to compete with oil refineries. One of the ways to produce biolubricants is through double transesterification from vegetable oils, where the role of catalysts (usually homogeneous) is vital, as they can improve the yield of the process. However, they should be removed after the chemical reaction, which is difficult once the biolubricant is obtained. Otherwise, they could act as catalysts during oxidation, contributing to a further decrease in oxidation stability and provoking significant changes. To avoid this, antioxidant addition could be an interesting choice. The aim of this work was to assess TBHQ addition in frying oil biolubricants, monitoring properties such as viscosity, acid number, absorbance or TBHQ content (through voltammetry) during oxidation. TBHQ addition (2114 mg·L^−1^) kept the main quality parameters during oxidation compared to control samples. In contrast, TBHQ content decreased during oxidation (to 160 mg·L^−1^), which proved its antioxidant effect.

## 1. Introduction

### 1.1. Current Energy and Raw Material Shortage

Considering the current energy and raw material instability on account of geopolitical aspects (such as trade or real wars), further efforts are being made in order to move towards sustainable practices, such as the real and definitive implementation of renewable energies, green chemistry or a circular economy, among others. Thus, this transition could change or even reverse the currently established roles when it comes to geopolitics, as some authors have pointed out [1].

At present, there seem to be serious steps that are going to be taken, as opposed to the typical unfulfilled promises and fine words from government and international agencies. In that sense, and apart from the obvious (and vital) advantages related to the environment (reduced greenhouse gas emissions, low water, air and soil pollution, avoidance of acid rain, ecosystem conservation, etc.), there are more and more convincing economic considerations to go forward with green policies. Thus, the economic independence (with reduced import dependence and an increase in renewable energy resources) and the promotion of local economies (through raw materials or wastes related to renewable energy use) might be the definitive levers that can unlock this situation [2].

Regarding alternatives for the petroleum industry, biorefineries could play an important role, as many petroleum-based products can be replaced by natural products such as biodiesel or biolubricants, among others. Hence, biorefineries perfectly adjust to green chemistry, sustainability and a circular economy, as they tend to use natural raw materials (many of them wastes) in order to obtain a wide range of natural products (some of them reusable in the same process, with high atom economy levels), implying a lower environmental impact, as these products are usually biodegradable and their production less polluting [3,4,5]. Biolubricant production, depending on their chemical route, could contribute to the implementation of a biorefinery [6,7], as explained in the following subsection.

### 1.2. Biolubricant: A Real Alternative for Petroleum-Based Products

Biolubricants act as an alternative for conventional lubricants; that is, a thin layer between two surfaces to avoid friction and other undesirable effects such as erosion, degradation, corrosion, oxidation, etc. Thus, the role of biolubricants is vital, as they extend the lifetime of industrial equipment, whereas the environmental impact is not considerable compared to their petroleum-based equivalents [8]. One of the most interesting raw materials to obtain biolubricants are vegetable oils, which can contribute to the sustainable economic growth of developing countries as there are plenty of oil plants that can adapt to multiple and extreme climates or soils. Many studies have dealt with the possible production of biolubricants from vegetable oils such as cardoon, rapeseed, soybean, castor, sunflower or safflower oils, among others [9,10,11,12,13]. The use of the corresponding crops could present some advantages, as they can take part in crop rotation to keep soil quality. However, some challenges such as the possible competition with food crops should be considered. In that sense, the use of wastes such as frying oils (or waste cooking oils) could be interesting, as the management of these wastes might be complex and difficult. The valorization of frying oils to produce other products such as biodiesel or biolubricants is necessary, obtaining interesting and convincing results in the literature [14,15]. In this work, the use of frying oil from local homes and restaurants was selected.

Regarding biolubricant production, different chemical routes have been considered in the literature, with epoxidation and double transesterification being the most interesting ones [16]. Concerning the latter, it could imply the implementation of a biorefinery due to different reasons:

- This process uses raw materials such as vegetable oils.
- Many valuable products can be obtained, such as fatty acid methyl esters (FAMEs, biodiesel), glycerol (depending on its purity, it presents many uses), fatty acid esters (biolubricants) and methanol (which can be reused in the process, as explained later on).
- The atom efficiency in this process is high, as all the by-products can be directly used in industry or re-used in this process.

**Figure 1:**
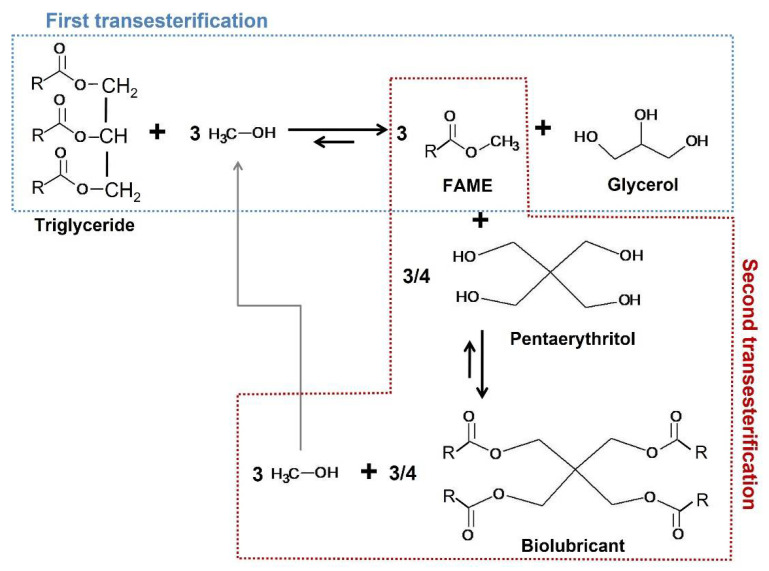
Biolubricant production through double transesterification.

This way, as explained in Figure 1, a first transesterification with fatty acids and methanol takes place, obtaining fatty acid methyl esters (FAMEs) and glycerol. Once these FAMEs are obtained and purified, they react with a more complex alcohol such as a polyol (for instance, pentaerythritol) in a second transesterification, to obtain the final biolubricant and methanol (which can be reused).

In order to make this process more efficient (and, therefore, competitive compared to a conventional refinery based on petrol compounds), the use of catalysts is vital, as they reduce the activation energy in both steps and, therefore, lower temperatures and reaction times are required. Finally, the main products obtained in this double transesterification (that is, biodiesel and biolubricant) present one challenge that should be solved: they usually present low oxidative stability values. Consequently, auto-oxidation processes can take place, with initiation (free radical generation), propagation and termination (generating stable products, different from initial biolubricants) stages [17]. This can result in an inconvenience, as these products can change their main physical–chemistry properties (with viscosity and acid number increase) during storage or oxidation. In this way, the fatty acid composition of the raw material (for instance, with a high linoleic/oleic ratio), along with other factors such as the presence of impurities (such as catalysts) in the final product could contribute to the decrease in oxidation stability [17]. As explained in the following sections, the use of antioxidants can be useful to avoid this problem.

### 1.3. The Role of Catalysts in Biolubricant Production: Yield and Quality

As previously explained, biorefineries (or any sustainable process that pursues the replacement for pollutant practices) need to be as efficient as possible, so that green transition is competitive. In such a way, the use of existing industrial facilities, cheap raw materials (even wastes that are easily obtained and have a complex environmental management) or catalysts (to improve the yield and efficiency of the process) are desirable. Concerning the latter, in the case of biolubricant production through double transesterification, both homogeneous and heterogeneous catalysts can be used. The former (including sodium or potassium methoxide or hydroxide) are more popular and common for biodiesel and biolubricant production, but they need to be removed through different processes such as washing treatments. The latter do not present that disadvantage (they are easily removed through filtration), although there are many challenges such as their low reusability or efficiency, mainly due to leaching [18,19]. The use of homogeneous catalysts is common in biodiesel production through transesterification, without leaving any traces in the final product, whereas biolubricants can present, after the use of these catalysts, some traces in the final product (as washing is not recommended to avoid hydrolysis).

Apart from the fatty acid composition of the raw material, these traces (normally Na^+^ or K^+^, derived from their corresponding hydroxides or methylates, among others) can contribute to oxidation during storage, accelerating the degradation of biolubricants (which mainly implies changes in viscosity and acid number, making their marketability difficult). In other words, the trace of catalysts could decrease (even more) the low oxidative stability that vegetable oil biolubricants usually present [17].

Nevertheless, there are different alternatives to avoid the further decrease in oxidation stability due to catalyst addition:

- Heterogeneous catalysts. Although they present the abovementioned disadvantages, it is an interesting field with room for improvement.
- The use of mild reaction conditions. As previous studies pointed out, the use of a vacuum in biolubricant synthesis (when possible) to increase the yield of the process at the expense of lower catalyst content and temperature could contribute to the increase in oxidative stability [20].
- Antioxidant addition. This is one of the most popular steps to improve the oxidative stability of biodiesel and biolubricants, which could avoid the contribution of a fatty acid profile (high linoleic/oleic acid in raw materials, for instance) and catalyst content in the final product [17].

In this work, we will pay attention to the latter option, which will be explained in the following subsection.

### 1.4. Antioxidants to Improve Oxidative Stability in Biolubricants

In order to improve the oxidative stability of biofuels and bioproducts such as biodiesel and biolubricants, different antioxidants have been widely studied in the literature, mainly applied to biodiesel. They can act in different ways to avoid or delay oxidation in the abovementioned products, mainly through scavenging, chelation or cutting off the auto-oxidation chain reaction, among others [17]. There are both natural and synthetic antioxidants, with the latter being more effective in increasing oxidation stability. Nevertheless, the use of natural antioxidants seems promising as they can contribute to sustainability, recently obtaining more and more effective results in the literature, in many occasions complying with standards at relatively low concentrations (around 1000 ppm) [21,22]. Concerning synthetic antioxidants, one of the most popular products are phenolic compounds. For instance, butylated hydroxytoluene (BHT), butylated hydroxyanisole (BHA), propyl gallate (PG) or tert-Butylhydroquinone (TBHQ) have presented high effectiveness increasing the oxidative stability of biofuels and biolubricants. Depending on the product or the combination with other additives, the efficiency of these antioxidants might vary [23]. In general terms, TBHQ is one of the most effective antioxidants concerning oxidation stability increase in biodiesel and biolubricants. Many studies have covered its efficiency, reaching long oxidation stability values in biodiesel (exceeding 8 h, the lower limit included in the UNE-EN standard) at low concentrations (normally from 1000 to 1500 ppm) [24,25,26], whereas previous studies have pointed out the importance of this product in keeping some basic properties (altered during oxidation) such as viscosity or acid number in biodiesel [25,27] and biolubricants (based on trimethylol propane or pentaerythritol, among others) [28] from different sources, such as cardoon, frying, rapeseed or safflower oils. This way, TBHQ could be another interesting parameter to be monitored during biodiesel or biolubricant storage or oxidation, as its decrease in content could provide information about the right amount of antioxidant that should be used, or the service life of these products. As explained in the following section, voltammetry could be a useful way to determine TBHQ evolution in biofuels or biolubricants.

### 1.5. Voltammetry as a Suitable Technique to Determine Antioxidant Content

The use of voltammetry (as an electroanalytical method to obtain information about an analyte by measuring the current as the potential) has been widely studied in the literature, specifically when it comes to TBHQ determination in some mediums such as biodiesel. There are two kinds of voltammetry: cyclic voltammetry (CV) and differential pulse voltammetry (DPV):

- CV: In this case, a linear potential scan (with a triangular shape) as a function of time is carried out, from an initial potential (E_i_) to a certain final potential (E_f_) and, afterwards, the scan is usually inverted to E_i_. The signal is obtained when a stationary electrode is immersed in a chemical solution without stirring. CV is used to carry out studies about electrochemical reaction mechanisms, electrode processes or organic compounds. Additionally, it is used for quantitative purposes, presenting lower sensitivity values (LOD 10^−5^ M) than other voltammetric techniques.
- DPV: It is applied as a function of potential over time, and the excitation signal consists of a series of pulses (shaped like stairs), where the base potential gradually increases at small intervals between 5–10 mV. The signal is measured as the difference between the intensity obtained before the pulse and the one obtained before the end of the pulse application. That provokes a decrease in the capacitive current, obtaining a higher sensitivity (LOD 10^−7^ M).

The most important parameters in CV are the anode and cathode peak potentials (E_ap_ and E_cp_, respectively), as well as the corresponding intensity values (I_ap_ and I_cp_). Regarding DPV, the most important parameters are I_p_ and E_p_, which are used to obtain the peak height; an interesting parameter to determine the antioxidant contentas it is proportional to the antioxidant concentration in samples.

As previously explained, this technique could be useful to determine TBHQ content in biolubricants. Indeed, previous studies have covered electroanalytical methods applied to TBHQ determination in similar products, such as biodiesel. De Araújo et al. studied an electroanalytical method to determine TBHQ in soybean biodiesel, applying surfactant species and obtaining an oxidation peak (which was not well-defined) for TBHQ [29,30].

On the other hand, Tormin et al. used an amperometric determination for TBHQ in biodiesel, adding an aliquot of ethanol:water (75:25) and applying three sequential potential pulses, comparing these results with HPLC [31]. Caramit et al. developed a method to simultaneously determine TBHQ and BHA in biodiesel through a voltammetric method [32].

Goulart et al. used DPV with a glassy carbon electrode to determine TBHQ in biodiesel. Previously, the samples underwent a liquid–liquid extraction with different solvents (acetonitrile and ethanol), obtaining TBHQ recoveries of 110 and 99%, respectively [33]. Squissato et al. used square wave voltammetry (SWV) with a gold electrode to determine copper (which plays an important role during oxidation) and TBHQ, obtaining recoveries of 97 and 104%, respectively [34].

Regarding our previous works, we have successfully used voltammetry to determine TBHQ in different samples (such as cardoon, rapeseed or safflower biodiesel) during oxidation [25], implying an interesting background to adapt this method to TBHQ determination in biolubricants, where some conditions such as viscosity and composition can be different.

### 1.6. Novelty and Aim of This Work

To the best of our knowledge, and even though there are some works dealing with biolubricant production through different chemical routes, some of them including frying oil (or waste cooking oil) as a raw material, the specific use of a frying oil biolubricant obtained from double transesterification with pentaerythritol has not been widely covered in the literature. In addition, the effect of oxidation on these samples has not been studied to a large extent and, in this work, we include interesting parameter studies such as UV-absorbance. Finally, the use of voltammetry to determine the TBHQ content (and its evolution during oxidation) in biolubricants is an innovative technique that needs to be adapted to the particularities related to these products (especially the high viscosity value compared to biodiesel, where this technique has been studied in our previous works [25]).

Considering the above, the aim of this work was to study different quality parameters (including TBHQ addition through voltammetry) during oxidation in a biolubricant based on waste cooking oil, obtained through double transesterification with methanol and pentaerythritol, to assess its suitability for industrial use.

## 2. Results and Discussion

### 2.1. Biodiesel Characterization

Regarding frying oil biodiesel (FOBD) production, its main characteristics are included in Table 1.

**Figure 2:**
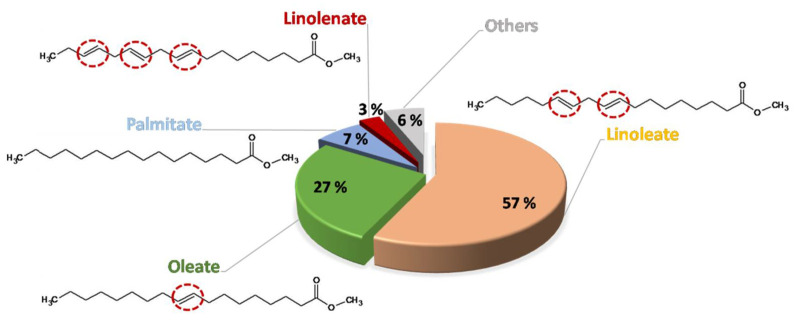
Fatty acid methyl ester profile of frying oil biodiesel (FOBD). Double bonds are circled in red.

As observed, most parameters were within the standard, including a high FAME content (that is, a high reaction yield for the first transesterification) and high flash and combustion points, which imply safety during storage. The acid number and moisture were low, which is recommendable for a good performance in diesel engines, and the cold filter plugging point (CFPP) and viscosity values were within the optimum range, which is vital to avoid obstructions in diesel engines. However, even though the sample had a low iodine value (IV, which could indicate low double bond content and the possibility of longer oxidative stability, as IV is related to the oxidation trend of biodiesel [35]), the oxidation stability was short, not achieving 3 h, which could be due to the FAME profile included in Figure 2.

Thus, the FAME profile, highly related to the fatty acid profile of raw materials (as the conversion of fatty acids to fatty acid methyl esters is high and similar for each kind of compound), plays an important role in oxidation stability, especially concerning double bonds and conjugated double bounds [36]. This way, double bonds (and, especially, conjugated double bonds) are usually the starting point of free radicals during oxidation. Therefore, the more double bonds there are, the lower oxidation stability the sample presents. Consequently, the FAMEs included in this figure could be ordered by oxidative stability, from short to long, such as the following: methyl linolenate, linoleate, oleate and palmitate. Due to the fact that FOBD presented a high percentage of methyl linoleate (57% in this case) and considering the low percentage of methyl palmitate (7%) and methyl oleate (27%), the short oxidation stability could be justified. Nevertheless, other biodiesel samples, coming from the same raw material or presenting a similar fatty acid profile, had similarly low oxidation stabilities [25].

### 2.2. Biolubricant Characterization

Concerning the frying oil biolubricant (FOBL), the main characteristics are included in Table 2.

It should be pointed out that the high conversion obtained (exceeding 90%) is similar to previous works and other works included in the literature [8,28,37].

As expected, most characteristics differed from the original properties of FOBD, due to the change in the molecular structure on account of the use of pentaerythritol (instead of methanol) for transesterification. This way, viscosity at 40 °C considerably increased from 4.59 to 68.5 mm^2^·s^−1^ for the FOBD and FOBL, respectively. This could be due to the molecular complexity of the FOBL, as observed in Figure 1, implying more intermolecular interactions such as hydrogen bonds or van der Waals force. Consequently, viscosity (that is, resistance to flow) increases, because it is directly related to these interactions, as explained elsewhere [38]. In addition, viscosity at 100 °C exceeded 10 mm^2^·s^−1^, with a viscosity index of 144, which is an indicator of viscosity changes over temperature. Thus, the higher the viscosity index is, the lower the changes in viscosity (which is desirable). As observed in the literature, these values were equivalent to other biolubricants similarly obtained with polyols such as pentaerythritol or trimethylolpropane, among other vegetable or mineral oils, ranging from 127 to 226 [7,16,28,39]. Additionally, flash and combustion points were higher with the subsequent positive impact on safety during storage, consistent with other results found in the literature, with minimum values of around 250 °C for different vegetable oils and pentaerythritol or trimethylolpropane-based biolubricants [16].

However, other properties were highly influenced by the raw material and the intermediate product obtained during double transesterification (that is, biodiesel).That was the case for oxidation stability, which was similar (although slightly shorter) to the value obtained for FOBD. Thus, the same explanation given for FOBD could be suitable for the low oxidation stability of FOBLs. As explained in other studies, depending on the nature of the raw material, biolubricants could present different oxidation stability values [40]. Additionally, there are two factors that could further reduce this value: first, the product (FOBD) underwent an additional step, with a high reaction temperature (160 °C, as explained in the Section 3), which could contribute to the deterioration of the sample; second, during the purification of the FOBL, washing treatment was not used (to avoid hydrolysis), and some traces of catalysts could have remained in the sample, promoting the auto-oxidation of the final biolubricant obtained. Previous studies pointed out this fact, promoting the use of lower temperatures and lower amounts of catalysts [20].

Therefore, both FOBD and FOBLs needed the addition of antioxidants such as TBHQ, which will be accomplished in the following subsection.

### 2.3. TBHQ Addition to Biodiesel and Biolubricant

**Figure 3:**
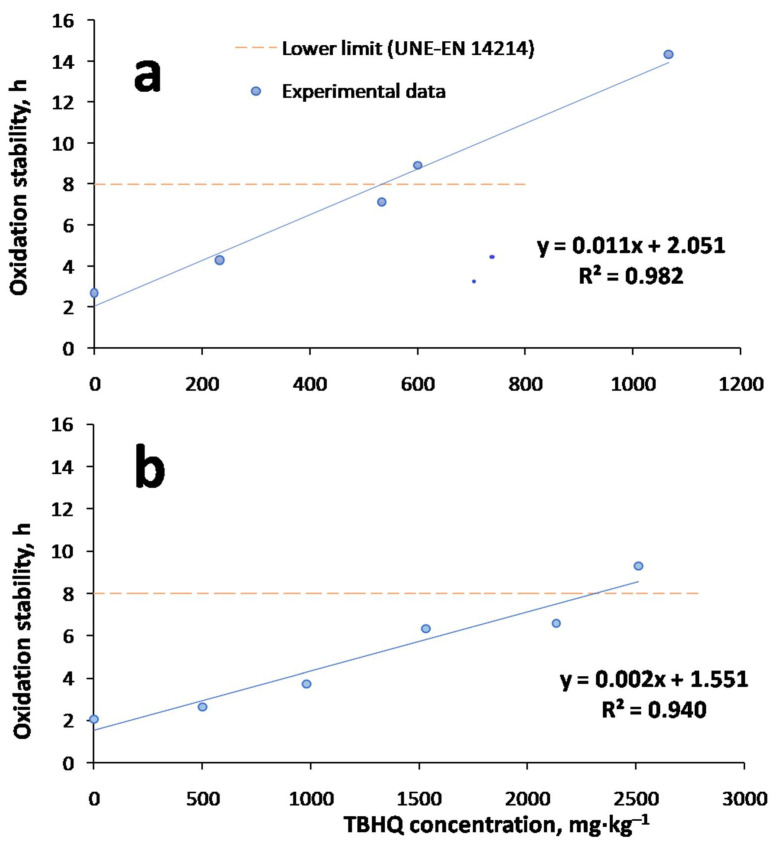
TBHQ addition and its effect on oxidative stability of (**a**) FOBD and (**b**) FOBL.

As explained in the previous subsection, the addition of TBHQ was justified according to the low oxidation stability values obtained. Thus, antioxidant addition is essential to avoid changes in viscosity or acid number, among other factors, during oxidation or storage. In order to determine the optimum TBHQ addition on FOBD and FOBLs, Figure 3 shows the effect of TBHQ on the oxidative stability of FOBD and FOBLs.

As observed in both cases, there was an increase in oxidation stability with TBHQ concentration, and this trend showed a good adjustment of the experimental data to a regression line. Thus, in the selected concentration range (0–2500 mg·kg^−1^), a linear trend was observed. Other authors stated that these trends might vary at high antioxidant concentrations, presenting a plateau or even a decrease in oxidative stability, with the consequent influence of antioxidant concentration on oxidative stability [17].

Regarding FOBD, the optimum TBHQ concentration (Op. TBHQ) to comply with the UNE-EN 14214 standard (that is, the lower limit of 8 h) was 565 mg·kg^−1^ (519 mg·L^−1^), whereas in the case of the FOBL, this value was 2300 mg·kg^−1^ (2114 mg·L^−1^). Even though this standard is not applied to biolubricants, we took the lower limit of 8 h as a reference for the final biolubricant to establish a comparison between FOBD and FOBLs. This way, comparing both optimum TBHQ concentrations, it was higher in the case of the FOBL, which could be due to the lower oxidative stability observed in the previous section, requiring more TBHQ concentration to surpass the lower limit of 8 h.

Consequently, the optimum concentration of TBHQ for the FOBL (Op. TBHQ = 2300 mg·kg^−1^ or 2114 mg·L^−1^) was selected for oxidation experiments, included in the following subsections.

### 2.4. TBHQ Determination through Voltammetry

With the aim of quantifying TBHQ in FOBLs, previous studies have been carried out using different electroanalytical techniques (cyclic and differential pulse voltammetry, CV and DPV, respectively) and different mediums (emulsion and dispersive microextraction, EMUL and MED, respectively). Thus, the aim was to select the most suitable technique and medium, according to quickness, easiness, reproducibility, etc.

TBHQ quantification in FOBLs will cover doped samples with different amounts of antioxidant, as well as FOBL with the optimum TBHQ concentration (Op. TBHQ), undergoing different oxidation times.

#### 2.4.1. Previous Studies

**Figure 4:**
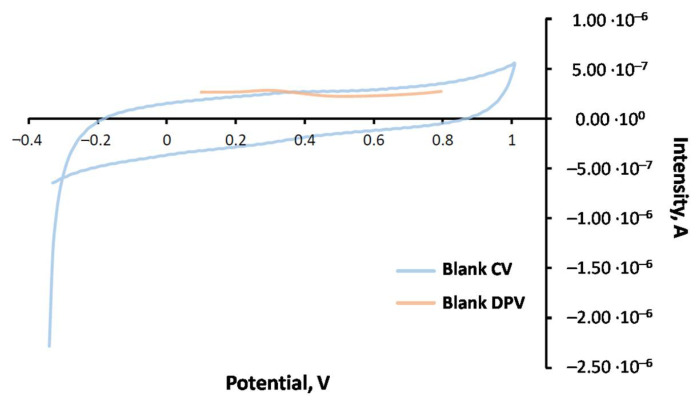
CV and DPV voltammograms for blank samples.

In order to select the right technique and medium for TBHQ analysis in FOBLs, some previous studies were carried out. Thus, the FOBL was doped with the corresponding amount of TBHQ to obtain a concentration of 2114 mg·L^−1^. Once obtained, the sample was prepared in emulsion (EMUL) and dispersive microextraction (MED) mediums, recording the corresponding voltammograms through CV and DPV. For this purpose, a glassy carbon electrode (GC) was used, requiring its cleaning with dimethylformamide (DMF) and ultrapure water between measurements. This is an important step to avoid any remains of FOBL on the electrode surface after recording. To check if the electrode is clean, a blank (containing ethanol, 8 mL; buffer, 4 mL and pH = 2.5; and CTBA, 0.8%; diluting with ultrapure water to 50 mL) was registered. Figure 4 shows a typical voltammogram for these cases.

**Figure 5:**
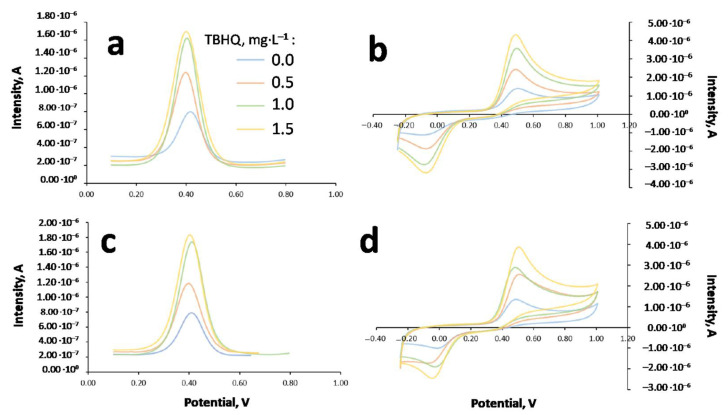
Voltammograms obtained for FOBL with TBHQ (2114 mg·L^−1^) with some standard additions using different extractions and voltammetries. (**a**) Dispersive microextraction, DPV; (**b**) dispersive microextraction, CV; (**c**) emulsion medium, DPV; (**d**) emulsion medium, CV.

Table 3 shows the main results obtained for TBHQ quantification in the biolubricant for both mediums through standard addition (reference standard = 498 mg·L^−1^ in MeOH), and Figure 5 shows the corresponding voltammograms for the different techniques and mediums.

It can be observed that, both in emulsion and dispersive microextraction mediums, CV shows an oxidation response for TBHQ at around 0.5 V, whereas in the reverse scan, its reduction response was at 0.0 V. Regarding DPV, an oxidation signal at 0.42 V was observed. The intensities obtained in both mediums for each technique were similar. Therefore, sensitivity was similar for each technique regardless of the medium. Further, it can be noted that initial intensities were higher in the CV sample. However, the detection limit was higher for CV (10^−5^ M) compared to DPV (10^−7^ M). In spite of this higher detection limit, CV presents an interesting advantage, such as the fast scan speed (150 mV·s^−1^, which was used in this study). Consequently, the residence time of the sample was short, reducing electrode poisoning and making the cleaning process easier. That is the reason why CV was selected as the voltammetric technique for further experiments.

**Figure 6:**
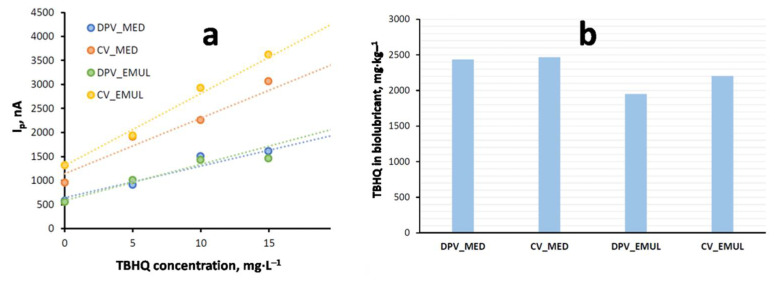
Differences between CV and DPV through emulsion medium (EMUL) and dispersive microextraction (MED): (**a**) changes in intensity peak; (**b**) calculated TBHQ concentrations.

Figure 6a shows the variation of peak intensities for CV and DPV in an emulsion medium (EMUL) and a dispersive microextraction medium (MED) depending on the added TBHQ concentration (according to standard addition). On the other hand, from the equations corresponding to standard addition lines, TBHQ concentration through CV and DPV was calculated, as can be seen in Figure 6b. As observed, the results obtained through microextraction were similar for CV (2435 mg·L^−1^) and DPV (2466 mg·L^−1^), whereas in the emulsion medium, the results considerably differed, from 1950 mg·L^−1^ for DPV to 2204 for mg·L^−1^ CV.

In the following sections, other experiments at different TBHQ concentrations (588 and 1044 mg·L^−1^) using EMUL and MED will be included, selecting CV as the voltammetry technique.

##### Emulsion Medium in CV

**Figure 7:**
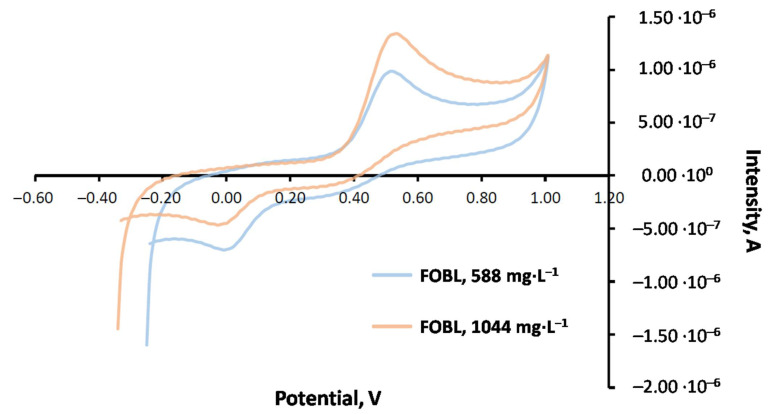
Cyclic voltammograms (150 mV·s^−1^) obtained in emulsion medium for FOBL at different TBHQ concentrations (588 and 1044 mg·L^−1^). Final concentrations in the cell: 11.76 and 20.88 mg·L^−1^, respectively.

TBHQ quantification in the FOBL was carried out in an emulsion medium through CV and standard addition, at two different concentrations (588 and 1044 mg·L^−1^). For this purpose, the FOBL (25 mL) was doped with the corresponding amount of TBHQ standard, undergoing ultrasound for 5 min. Afterwards, the corresponding voltammograms were recorded twice. The results are included in Figure 7.

From the data corresponding to the calibration line, the concentrations of the doped FOBL (with 588 and 1044 mg·L^−1^ TBHQ) were calculated. Table 4 shows these results.

##### Liquid–Liquid Dispersive Microextraction in CV

**Figure 8:**
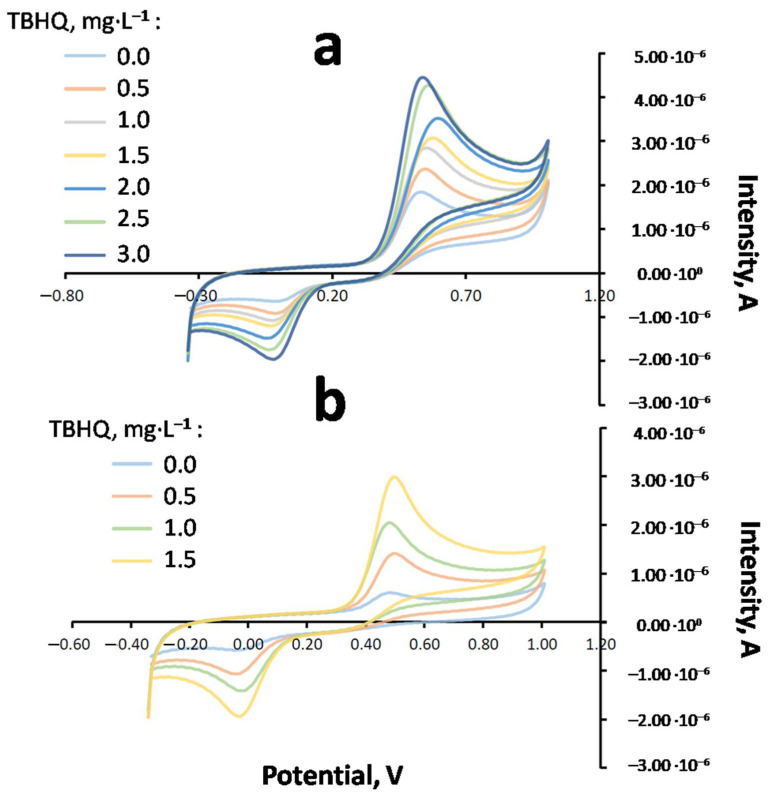
Cyclic voltammograms with standard additions (498 mg·L^−1^ TBHQ) for FOBL with TBHQ (1044 mg·L^−1^) through (**a**) emulsion medium and(**b**) dispersive microextraction.

Previous studies were carried out through liquid–liquid dispersive microextraction to quantify TBHQ in FOBLs. In this case, the sample was doped with TBHQ (1044 mg·L^−1^), using the standard addition (TBHQ 498 mg·L^−1^ in methanol). Figure 8 shows a comparison between the emulsion medium and dispersive microextraction for the FOBL doped with TBHQ, including some standard additions:

Thus, Table 5 shows the main results for TBHQ quantification in the doped FOBL (1044 mg·L^−1^) through dispersive microextraction and CV.

As observed, cyclic voltammetry through liquid–liquid dispersive microextraction had some advantages compared to the emulsion medium. Among them, the more effective cleaning of the glassy carbon electrode should be noted. In addition, reproducibility has higher, as microextraction removes some components in biolubricants that can promote the addition of TBHQ to the electrode surface. For this reason, this working medium was selected to quantify this antioxidant.

##### Influence of Ethanol Content on Liquid–Liquid Dispersive Microextraction in Cyclic Voltammetry

**Figure 9:**
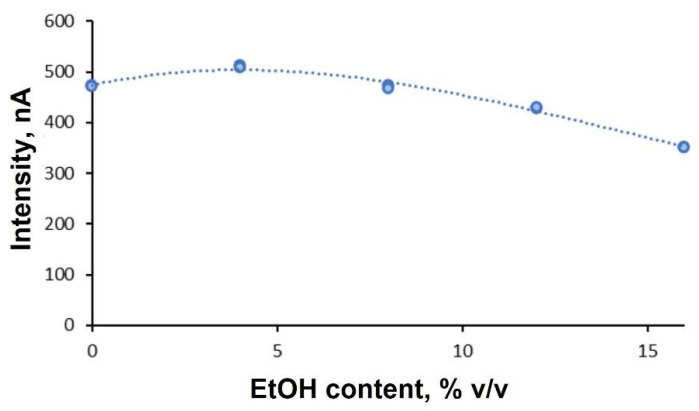
Influence of ethanol on the sample through dispersive microextraction.

On the other hand, the influence of ethanol percentage in the final solution was assessed, carrying out five measurements in samples with different ethanol volumes (0, 2, 4, 6 and 8 mL). As observed in Figure 9, there was no significant influence on intensity when different volumes of ethanol were added. Therefore, we decided to prepare further samples by adding 8 mL of ethanol.

#### 2.4.2. TBHQ Analysis in FOBL with the Optimum Antioxidant Addition, Undergoing Different Oxidation Times

**Figure 10:**
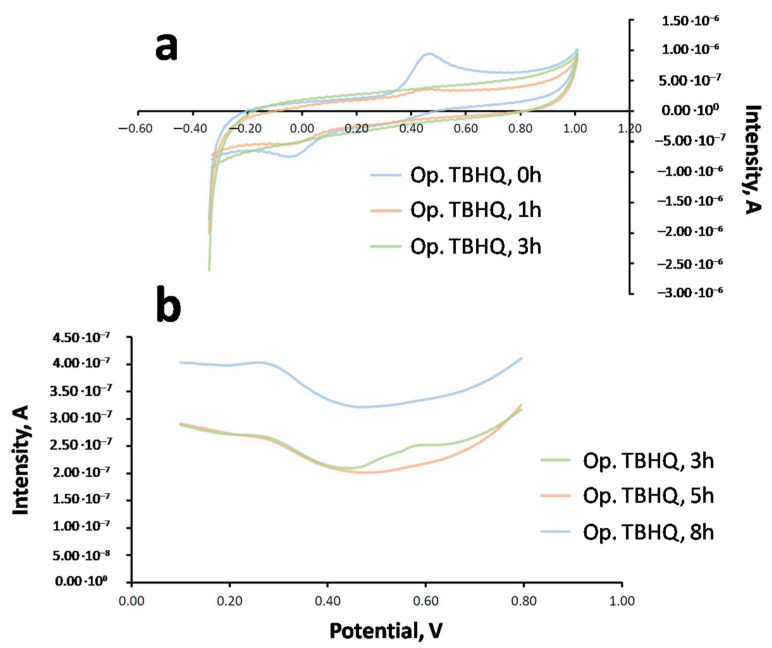
Voltammograms obtained through CV and dispersive microextraction for FOBL (Op. TBHQ) at different oxidation times: (**a**) CV at 0, 1 and 3 h; (**b**) DPV at 3, 5 and 8 h.

Once the voltammetric technique and medium were selected, the analysis of the optimum amount of TBHQ (2300 mg·kg^−1^ or 2114 mg·L^−1^) included in the FOBL to keep an oxidative stability of 8 h was carried out. After cleaning the electrode, TBHQ was determined through standard addition using the TBHQ standard (498 mg·L^−1^), as previously explained. Figure 10 shows some voltammograms for Op. TBHQ at different oxidation times. It can be observed that, for 0, 1 and 3 h (see Figure 10a), the oxidation signal decreased with the oxidation time. Moreover, after 3 h or oxidation, the oxidation signal was not observed through CV due to the lower sensitivity of this technique (detection limit of 10^−5^ M). For this reason, in order to completely monitor TBHQ content throughout the oxidation test (up to 8 h), DPV was selected (detection limit of 10^−5^ M), showing some results in Figure 10b.

**Figure 11:**
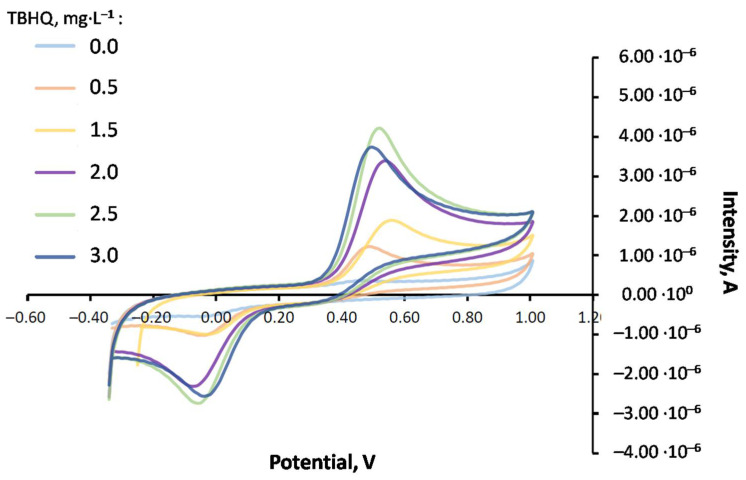
Cyclic voltammograms with different standard additions obtained through dispersive extraction for FOBL and Op. TBHQ (2114 mg·L^−1^) after 1 h of oxidation.

Additionally, Figure 11 is shown as an example where different CV voltammograms of FOBLs with the optimum amount of TBHQ (addition standard method with TBHQ standard of 498 mg·L^−1^) are included, after 1 h of oxidation.

As the use of a different technique (DPV) was necessary, in order to measure the TBHQ signal in FOBLs after 3 h of oxidation, TBHQ quantification at 0 h was carried out for CV and DPV through the standard addition method to check if the results were concordant. As a result, previous studies showed that both techniques could be used at high TBHQ concentrations [25].

Thus, the final results of the evolution of optimum TBHQ content in FOBLs during oxidation are included in the following section, where other parameters equally influenced by oxidation (such as viscosity or acid number) were included.

### 2.5. Effect of Extreme Oxidation on FOBL and Comparison with TBHQ Addition

Once the optimum concentration of TBHQ (Op. TBHQ = 2300 mg·kg^−1^ or 2114 mg·L^−1^) for the FOBL was calculated, the sample underwent extreme oxidation conditions, as explained in the Section 3. In order to compare control samples (without any TBHQ addition) and doped samples (that is, Op. TBHQ, with the optimum concentration of TBHQ) during oxidation, several parameters were measured at different oxidation times (0, 1, 3, 5 and 8 h) such as viscosity, acid number and UV absorbance.

#### 2.5.1. Effect of Extreme Oxidation on Viscosity

**Figure 12:**
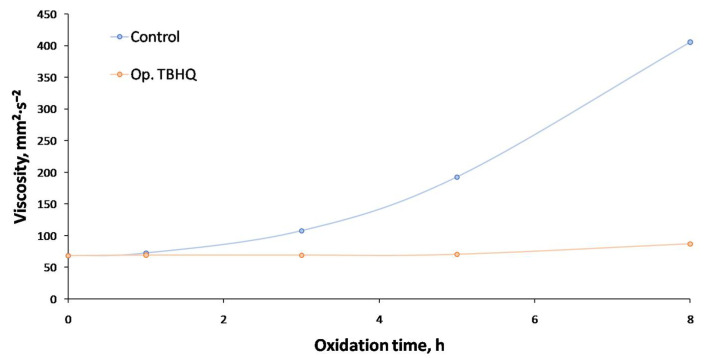
Effect of TBHQ addition on FOBL viscosity during oxidation.

During extreme oxidation (see Figure 12), both the control and Op. TBHQ samples presented an increase in viscosity. Nevertheless, this increase was considerably stronger in control samples, especially after 2 h of oxidation, from 68 to 405 mm^2^·s^−1^, whereas this increase was reduced in the case of TBHQ addition (from 68 to 87 mm^2^·s^−1^). In the case of the control samples, the oxidation process took place with a pronounced free radical generation, and subsequently the propagation and termination processes (which usually implies polymerization and, therefore, an increase in viscosity). When TBHQ was added, this oxidation process was slowed down, with a slower increase in viscosity. Previous studies pointed out the same behavior, as explained for biolubricants from high-oleic safflower oil through double transesterification with methanol and pentaerythritol, where a considerable increase in viscosity was equally observed during the oxidation of the control samples, whereas the doped samples kept their viscosity during the whole process [28].

Consequently, and comparing both trends, it could be said that FOBLs with TBHQ kept their viscosity during oxidation, especially up to 5 h. In the case of the control samples, there was a significant increase in viscosity, making the sample useless for their initial or specific use as a biolubricant.

#### 2.5.2. Effect of Extreme Oxidation on Acid Number

**Figure 13:**
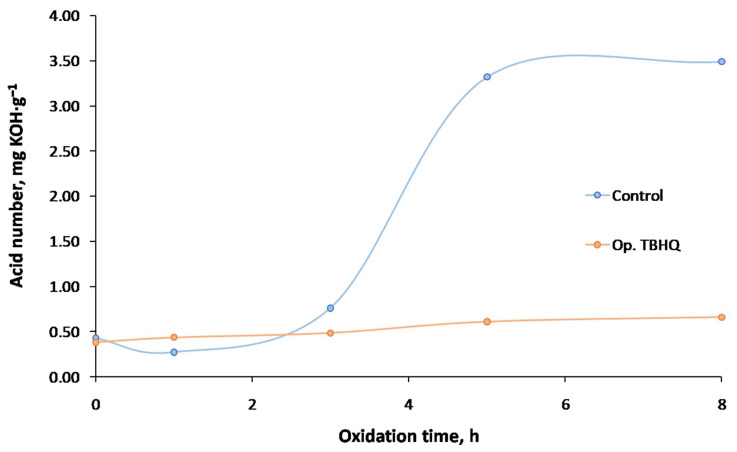
Effect of TBHQ addition on FOBL acid number during oxidation.

Regarding the acid number (Figure 13), the control samples had a considerable increase in this parameter after 3 h of oxidation, achieving 3.49 mg KOH·g^−1^ at 8 h. In the case of the doped samples (Op. TBHQ), there was a slight and constant increase in acid number, from 0.38 to 0.62 mg KOH·g^−1^ at 8 h. Thus, the generation of free fatty acids, among other degradation products derived from oxidation, was reduced when the antioxidant was added, compared to the control samples. The same trend was observed in other studies, where the use of TBHQ (1200 ppm) practically maintained the acid number of corn biodiesel during oxidation for 8 h, whereas in control sample, this value doubled [27]. For a biolubricant based on high-oleic safflower and pentaerythritol, an increase from 0.5 to 4.0 mg KOH·g^−1^ after 8 h of oxidation was observed, whereas with the use of TBHQ, this increase was negligible [28].

Consequently, the use of TBHQ is advisable to avoid a rapid increase in the acid number, which could be an undesirable effect during storage or its use in engines or machineries (whose useful life could be compromised).

#### 2.5.3. Effect of Extreme Oxidation on UV Absorbance

**Figure 14:**
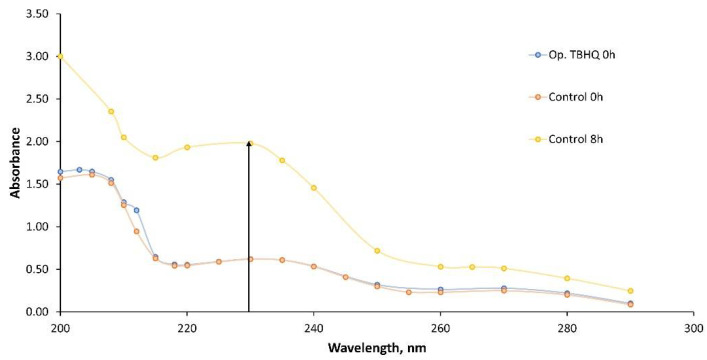
Wavelength selection to assess changes in UV during FOBL oxidation.

In order to determine the main changes in UV absorption, an initial scanning in the UV range (from 200 to 290 nm) was carried out, as observed in Figure 14. Thus, after 8 h of oxidation, control samples presented a different absorption profile compared to the initial sample, possibly due to the molecular changes that took place in oxidized samples, whose degradation products could alter the absorption in the UV range with the presence of chromophore groups. On the other hand, it should be noted that the optimum TBHQ addition did not practically alter the UV profile of the FOBL, possibly due to its low concentration in the final sample.

As a consequence, during oxidation, a considerable peak with a maximum at 230 nm appeared, selecting this wavelength for further studies (regarding the evolution of absorption at 230 nm during oxidation).

**Figure 15:**
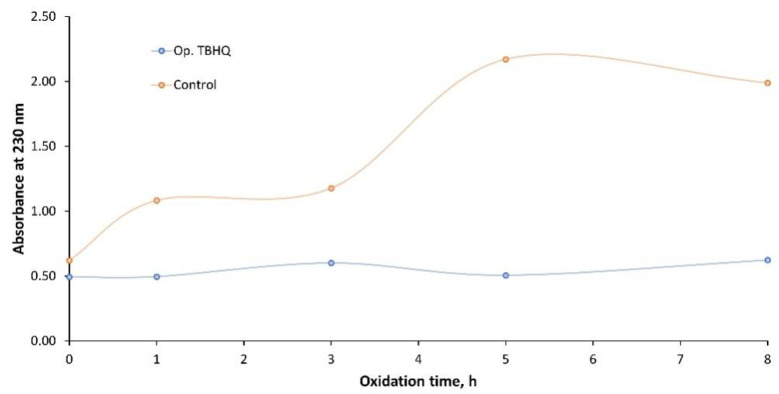
Effect of TBHQ addition on FOBL absorbance in UV during oxidation.

As such, and taking into account the abovementioned maximum peak at 230 nm, Figure 15 shows the main changes comparing the control and Op. TBHQ samples. Again, the control samples showed a considerable increase in absorbance, from 0.62 to 1.99, whereas in the case of doped samples, the increase was lower, from 0.51 to 0.62.

**Figure 16:**
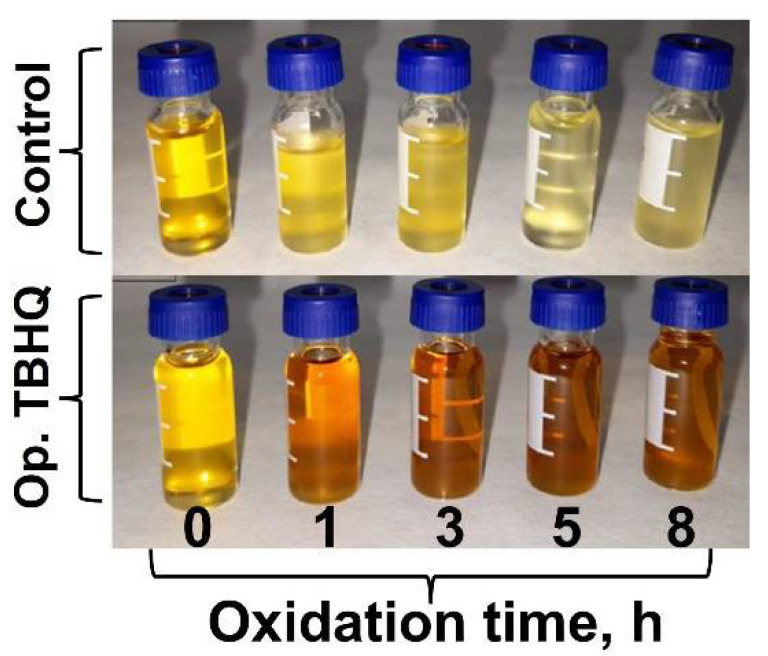
Visual changes during oxidation of FOBL.

Again, the properties of the FOBL were kept by adding TBHQ, possibly due to the fact that the molecular composition of the biolubricant was not altered during oxidation, avoiding the generation of degradation products which could alter the absorption in the UV range. In addition, visual changes were observed in the samples, as observed in Figure 16. Thus, two different behaviors were determined, with a decrease in color intensity and an increase in turbidity for the control samples and a continuous browning in the case of the doped samples. Regarding the control samples, these changes could be due to the degradation of fatty acid esters, generating more viscous products which could imply turbidity. For the doped samples, the browning effect could be due to the presence of TBHQ, which could provoke this change in color when it gets oxidized (instead of the final biolubricant). This behavior in polyphenols is typically observed in fruits and vegetables, where their presence can alter the visual quality of these products [41].

Consequently, and although there were visual changes both in treated and untreated FOBLs, the causes were different: on the one hand, visual changes were due to the degradation of the sample (which is undesirable), whereas on the other hand, these changes were on account of TBHQ oxidation, masking or interfering with the real color of the FOBL (which could be used as an indicator to assess the protective effect of antioxidants).

#### 2.5.4. Effect of Extreme Oxidation on TBHQ Content

**Figure 17:**
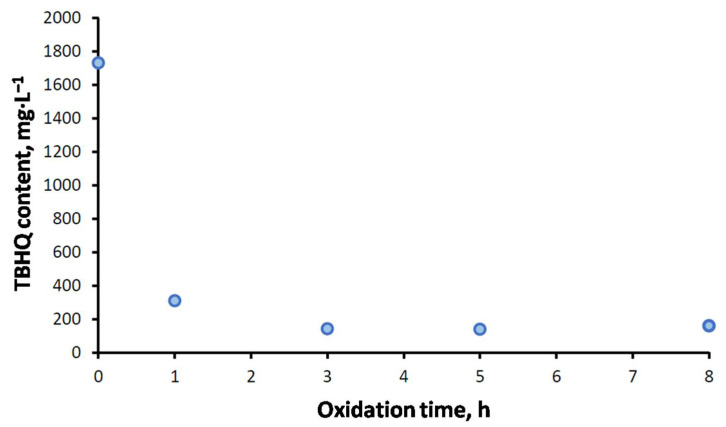
Effect of extreme oxidation on TBHQ content in FOBL.

As previously commented, the influence of oxidation could take place in FOBLs (especially when TBHQ was not added), but also in TBHQ (as expected, as it keeps FOBL properties at the expense of its own oxidation). Thus, in the previous subsection, the oxidation of TBHQ was pointed out according to the increase in browning in the sample. As a consequence, the oxidation of TBHQ should imply a decrease in the TBHQ content according to the analytical method used to assess it (that is, voltammetry), as the molecular structure of TBHQ (and, therefore, the detection through voltammetry) changes during oxidation. Figure 17 shows TBHQ evolution during the oxidation of doped samples. Equally, Table 6 shows the specific results for the TBHQ content at a specific oxidation time. As expected, there was a decrease in the TBHQ content, especially during the first hour of oxidation, where TBHQ content decreased from around 1800 to approximately 300 mg·L^−1^. At the end of oxidation, the amount of TBHQ was reduced, not reaching 200 mg·L^−1^. Previous studies on different biodiesel samples showed a similar trend, with a decrease in the TBHQ content (from their optimum value to comply with the standard of around 100 mg·L^−1^ in all cases), especially during the first two oxidation hours [25]. Consequently, the optimum amount of TBHQ selected for this study (2114 mg·L^−1^) was enough to avoid the depletion of the antioxidant, and this fact confirms the good performance of TBHQ at keeping the properties studied in this work.

To sum up, the main changes that took place in the control and doped samples at the end of the oxidation process are shown in Table 7.

As it can be inferred from this table, the changes in the control samples were considerable, quadruplicating the absorbance at 230 nm, with the viscosity and acid number showing a five-fold and seven-fold increase, respectively. These changes (that could equally take place during storage) would disable this biolubricant for any specific use in industry, as these products should present relatively stable values when it comes to viscosityto fulfill their specific role. Moreover, the high acid number would not be suitable for industrial use, to avoid corrosion or damage in industrial facilities. Nevertheless, thanks to the use of TBHQ, these changes (under extreme oxidation conditions) were not considerable compared to the control samples (around 20% in all cases), which points out the need for antioxidants to increase the oxidation stability of biolubricants. Regarding the TBHQ content, there was a 93% decrease after oxidation, which proved that the amount used in this experiment was enough to keep (to a certain extent) the properties of the biolubricant.

## 3. Materials and Methods

### 3.1. Raw Material

The raw material used in this study was frying oil (or waste cooking oil), which was collected from local restaurants and homes (in Badajoz, Spain) in 2021. The oil was filtered and its acidity was checked (below 1%, which allowed the process to continue without any further treatments). Afterwards, it was collected in 25-L containers at room temperature for further characterization and biodiesel and biolubricant production.

### 3.2. First Transesterification to Produce Fatty Acid Methyl Esters

**Figure 18:**
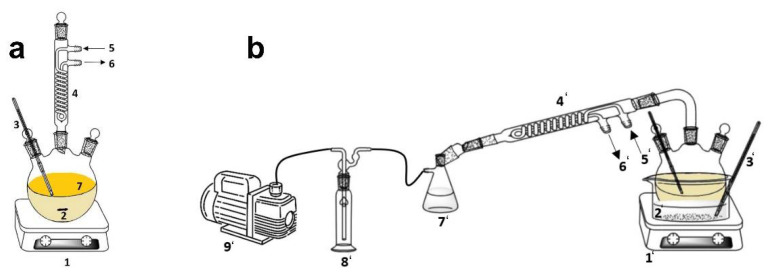
Experimental arrangement for (**a**) biodiesel production (1, heating system; 2, reactor; 3, temperature probe; 4, condenser; 5 and 6, water inlet and outlet; 7, reaction medium); (**b**) biolubricant production (1′, heating system; 2′, heating bath; 3′, temperature probe; 4′, condenser; 5′ and 6′, water inlet and outlet; 7′, methanol trap; 8′, trap flask; and9′, vacuum pump).

The chemical route to produce fatty acid methyl esters (biodiesel) was the transesterification of frying oil (fatty acids) with methanol. The experimental setup is shown in Figure 18a. The main chemical conditions to carry out this reaction were selected based on previous studies where a high-yield biodiesel was obtained [25,27]: methanol/oil ratio, 6:1; catalyst concentration (sodium methylate), 0.5% *w*/*w*; reaction temperature, 65 °C; stirring rate, 350 rpm; and reaction time, 120 min. Then, fatty acid methyl esters were separated from glycerol through decantation in a separating funnel and washing treatments with ultrapure water were carried out to remove the homogeneous catalyst, among other impurities. Finally, the sample was dried at 110 °C to avoid moisture and the final biodiesel (frying oil biodiesel, FOBD) was stored in 2.5-L opaque containers for further characterization and biolubricant production.

### 3.3. Second Transesterification to Produce Fatty Acid Esters

The chemical route to produce fatty acid esters (biolubricant) was a second transesterification of the abovementioned fatty acid methyl esters with pentaerythritol. This alcohol presents a more complex molecular structure compared to methanol, making transesterification more difficult and requiring higher temperatures or catalyst concentrations. The experimental setup included a trap for methanol collection, as methanol evolves during the process, as observed in Figure 18b. The main chemical conditions for this second transesterification are included in Table 8, based on previous studies where high biolubricant yields were obtained [28]:

The following steps were followed: frying oil biodiesel was heated in the reactor up to 100 °C, mixing it with the right amount of pentaerythritol (PE) and heating the blend to 160 °C. Then, sodium methylate (catalyst) was added, closing the system and starting the vacuum to obtain the expected pressure (260 mmHg). The reaction time started at this point, and the experiment took place for 2 h with the corresponding stirring rate to maintain a homogeneous reaction medium. Once the experiment ended, the sample was filtered through gravity and vacuum filtration (depending on difficulty), in order to remove surplus PE. The sample was filtered until a clear solution (not cloudy) was observed. Finally, the frying oil biolubricant (FOBL) was stored in 2.5-L opaque bottles for further characterization and treatments. It should be noted that traces of catalysts could be contained in the FOBL.

### 3.4. Antioxidant Addition

Once the FOBL was obtained, tert-Butylhydroquinone (TBHQ, 97%, Sigma-Aldrich, Saint Louis, MO, USA) was added to the samples to assess its effectiveness in improving the oxidative stability of the biolubricant. Different concentrations, typically studied in the literature for similar products such as fatty acid methyl esters (from 0 to 2500 mg·kg^−1^),were added to the FOBL [27,42,43]. Thus, the right amount of TBHQ was added to 100 mL of the sample, dissolving it by using ultrasound for 5 min. Afterwards, the samples were stored for further studies, such as oxidation stability determination through the Rancimat method. The optimum concentration to reach an oxidation stability of 8 h (taking the lower limit of the UNE-EN standard as a reference) was selected to undergo extreme oxidation conditions. 

### 3.5. Extreme Oxidation Conditions

Extreme oxidation conditions were selected to assess the evolution of four main characteristics of FOBLs, that is, viscosity, UV absorbance, acid number and TBHQ content (for those samples that were doped). These conditions were carried out according to previous studies (similar to the Rancimat method) [25,27], and the procedure was the following: 30 g of biolubricant was heated at 110 °C, bubbling synthetic air (100 L·h^−1^) for 8 h. During this process, samples at 0, 1, 3, 5 and 8 h were collected to measure their viscosity, UV absorbance, acid number and TBHQ content.

### 3.6. Sample Characterization

#### 3.6.1. Fatty Acid Methyl Ester Content and Composition

Fatty acid methyl ester (FAME) content and composition in FOBD (and FAME decrease during FOBL production) was measured through gas chromatography coupled with FID detection (Varian 3900, Agilent, Santa Clara, CA, USA).A polyethylene glycol column was used (Zebron ZB-WAX PLUS, Phenomenex, length: 30 m, film thickness: 0.5 µm and i.d.: 0.32 mm) and the main FAMEs determined were methyl oleate, linoleate, linolenateand palmitate. The internal standard used was methyl heptadecanoate.Thestandards were provided by Sigma-Aldrich (Saint Louis, MO, USA), and the chromatographic conditions were based on previous studies, according to the corresponding standard [44].

#### 3.6.2. Oxidation Stability

Oxidative stability (of FOBD or FOBL) was obtainedthrough the Rancimat method, as explained elsewhere [27,45]. Thus, 3 g of the sample was heated at 110 °C, bubbling synthetic air (10 L·h^−1^) and connecting the resulting stream of air to 50 mL of distilled water. Afterwards, the conductivity of water increased due to the dilution of the oxidation by-products in biodiesel or biolubricants, which was recorded with a conductivity meter (Mettler Toledo, Columbus, OH, USA). The oxidative stability, expressed in hours, was the time at which conductivity abruptly increased.

#### 3.6.3. Viscosity

In the case of FOBD, viscosity was obtained at 40 °C, whereas for FOBLs, viscosity was measured at 40 and 100 °C by using an Ostwald viscometer, following the corresponding standard [46]. These values were used to calculate the viscosity index, according to the standard [47].

#### 3.6.4. Acid and Iodine Number Determination

Acid and iodine numbers were determined according to the UNE-EN 12634:1999 and UNE-EN 14111:2003 standards, respectively [48,49]. Both parameters were analyzed for FOBD, whereas acid number was determined for FOBL, as this is one of the most changing parameters during oxidation or storage.

#### 3.6.5. UV Absorbance

In order to determine changes in UV absorbance, a UV-Visible spectrophotometer (UV-2005, J. P. Selecta, Barcelona, Spain) was used. The procedure was the following, based on previous works [50]: biolubricant samples (1 g) were dissolved in 300 mL of n-hexane (99.0%, PanreacApplichem, Barcelona, Spain). Afterwards, absorbance spectrums were determined within a specific wavenumber range (0–300 nm) for control samples and doped samples (with an optimum TBHQ content, as explained in previous sections) at different oxidation times (0, 1, 3, 5 and 8 h). Thus, the characteristic peak observed during oxidation (at 230 nm, as explained in the Section 2) was selected to observe the changes in absorbance taking place during oxidation.

#### 3.6.6. TBHQ Content through Voltammetry

TBHQ content in FOBLs by using cyclic (CV) and differential pulse (DPV) voltammetry was determined. For this purpose, voltammetric equipment (µAutolab, ECO Chemie, Utrecht, The Netherlands) was used, coupled with a 663 VA-Stand Metrohm unit (Herisau, Switzerland) with a three-electrode system: a glassy carbon electrode (as a working electrode), a reference electrode (Ag/AgCl) and an auxiliary electrode (Pt), with a measuring cell. The cleaning of the glassy carbon electrode between measurements consisted of rubbing it with cotton dipped in dimethylformamide (DMF) and cotton dipped in Milli-Q water. The TBHQ content in the biolubricant was determined as follows: in a 50-mL flask, 1 mL of sample (with the corresponding amount of TBHQ), 8 mL of ethanol, 4 mL of buffer (pH 2.5 NaH_2_PO_4_/H_3_PO_4_ 0.5 mol·L^−1^) and 2 mL of surfactant (0.8% cetyltrimethyl ammonium, CTAB) were added, and finally the solution was diluted to the mark with Milli-Q water, using ultrasound for 15 min to homogenize the final solution. Regarding statistical analysis, a statistical tool for analytical chemistry in the MATLAB 5.3 environment was used(ACOC V 2.0. program [51]), which carries out aunivariate calibration and quality parameter calculations for calibration lines.

Concerning the voltammetry of the sample, the following steps were carried out (in duplicate): First, the sample was introduced in the electrochemical cell, registering its cyclic voltammograms (150 mV·s^−1^). Afterwards, another sample with the same quantities, but adding suitable aliquots of TBHQ standard, was prepared for each experiment. For TBHQ quantification, the standard addition method was used, adding volumes of TBHQ standard and assessing each addition in duplicate and each standard addition in triplicate. On the other hand, the sample with the lowest TBHQ amount was also analyzed by using differential pulse voltammetry, obtaining the same results compared to CV.

For the emulsion medium (EMUL), 0.20 g of doped biolubricant was added to a 50-mL flask, where ethanol (8 mL), buffer (4 mL, pH = 2.5) and CTBA tensioactive (0.8%) were added, diluting with Milli-Q water. Afterwards, the sample underwent ultrasound for 2 min, and it was placed in the electrochemical cell (with previous de-oxygenation) with a nitrogen sweep for 2 min. Finally, the corresponding voltammograms were obtained.

Regarding liquid–liquid dispersive microextraction (MED), the sample was prepared as follows: 0.20 g of doped biolubricant was added to an Eppendorf tube, where 550 µL of extractant (1,4-dioxane) and dispersant (60:40 *v*/*v* ethanol/water solution, pH = 2.5) were equally added. Afterwards, the sample was agitated in vortex for 4 min, in order to separate the different phases. Then, the extract is obtained and added to a 50-mL flask, where ethanol (8 mL), buffer (4 mL, pH = 2.5) and CTBA tensioactive (0.8%) were added, diluting with Milli-Q water. Afterwards, the same procedure explained for the emulsion medium was followed.

#### 3.6.7. Other Quality Parameters

Moisture and density determinations were carried out according to the UNE-EN ISO 12937:2000 and UNE-EN-ISO 3675 standards, respectively. Concerning moisture, a Metrohm 870 trinitro plus equipment was used, and density was determined by using a pycnometer (Pobel, Madrid, Spain) [52,53].

The cold filter plugging point (CFPP)was determined through the EN 116 standard [54]. For flash and combustion points, the Cleveland open-cup method was used with the corresponding equipment (Herzog Cleveland semi-automatic, Herzog, Landa-Königshofen, Germany),according to the UNE 51-023-90 standard [55].

**Figure 19:**
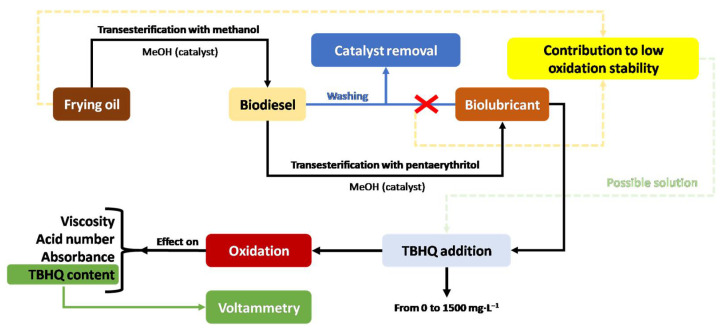
Experimental design for this work.

To sum up, the global scheme of the experimental design is included in Figure 19.

## 4. Conclusions

A summary of the main findings of this work is presented below:

- The use of frying oil as a biodiesel and biolubricant precursor was proved, obtaining for the former good quality parameters, most of them complying with the UNE-EN14214 standard. However, both the biodiesel and biolubricant had low oxidation stabilities, mainly due to the high linoleic/oleic ratio in the raw material. Furthermore, the lower oxidation stability found for the biolubricant was possibly due to the further treatment of the sample, as well as the presence of catalysts in the final product. In any case, the addition of TBHQ was necessary in order to improve this parameter and the subsequent storage period.
- TBHQ addition to frying oil biodiesel and biolubricants was effective, increasing their oxidative stability values up to 8 h with 519 and 2114 mg·L^−1^, respectively. With this concentration, the FOBL kept its properties (such as viscosity and acid number) undergoing extreme oxidation conditions. Thus, viscosity, acid number and absorbance increased 20% after 8 h of oxidation, whereas control samples showed considerable increases (500, 700 and 400%, respectively). TBHQ concentration decreased by 93% after extreme oxidation, proving that it was the optimum concentration for this biolubricant.
- Dispersive microextraction was used in order to quantify TBHQ in biolubricant, as it presented advantages in the emulsion medium such as easiness in glassy carbon electrode washing or higher reproducibility between samples. As a consequence, this electroanalytical method is proposed for TBHQ quantification in biolubricants, as it is simple, quick and low cost.
- The results obtained in CV or DPV were similar for each technique in both mediums. Thus, with microextraction, both techniques showed TBHQ concentrations around 2114 mg·L^−1^.
- The antioxidant content in doped samples (analyzed through CV or DPV) considerably decreased during oxidation, from 2114(doped samples at 0 h) to 160 mg·L^−1^ after 8 h, showing a drastic decrease from 1 h. This behavior pointed out the antioxidant activity of TBHQ, proving that the amount added to the biolubricant was enough for this experiment.

## Figures and Tables

**Table 1:** FOBD characterization and comparison with the standard.

Parameter	Units	Results	UNE-EN 14214 Limits	
			Lower	Upper
FAME content	%	97.10	96.5	n.a.*
Density	kg·m^−3^	886.30	860	900
Viscosity at 40 °C	mm^2^·s^−1^	4.59	3.50	5.00
Acid number	mg KOH·g^−1^	0.12	n.a.	0.50
Oxidation stability	h	2.68	8.00	n.a
Iodine number	g I_2_·100 g^−1^	99	n.a.	120
C. F. P. P. ^1^	°C	−1	−20	5
Flash point	°C	180	120	n.a.
Combustion point	°C	190	n.a.	n.a.
Moisture	mg·kg^−1^	230	n.a.	500

* Not applicable. ^1^ Cold filter plugging point.

**Table 2:** FOBL characterization.

Parameter	Units	Results
Conversion	%	92.60
Density	kg·m^−3^	919
Viscosity at 40 °C	mm^2^·s^−1^	68.5
Viscosity at 100 °C	mm^2^·s^−1^	10.64
Viscosity index	n.a. *	144
Acid number	mg KOH·g^−1^	0.44
Oxidation stability	h	2.07
Flash point	°C	253
Combustion point	°C	267
Moisture	mg·kg^−1^	600

* Not applicable.

**Table 3:** Intensity peaks (A) for TBHQ quantification in emulsion medium and dispersive microextraction for FOBL doped with optimum TBHQ concentration (2114 mg·L^−1^).

	Dispersive Microextraction		Emulsion Medium	
TBHQ, mg·L^−1^	DPV	CV	DPV	CV
0	581.7	960.0	555.2	1319.0
4.98	914.0	1913.0	1012.0	1938.0
9.96	1508.0	2263.0	1432.0	2933.0
14.94	1613.0	3069.0	1465.0	3625.0
19.92	1874.0	3266.0	2211.0	4222.0

**Table 4:** TBHQ concentration in doped FOBL through emulsion medium (588 and 1044 mg·L^−1^) through CV.

Replicates	Added TBHQ, mg·L^−1^	Recovered TBHQ, mg·L^−1^	Standard Deviation
2	588	417.25	±30
2	1044	887.50	±8

**Table 5:** TBHQ concentration in FOBL through dispersive microextraction (1044 mg·L^−1^).

Replicates	Recovered TBHQ (mg·L^−1^)
2	865.0
2	999.0
Standard deviation	±81

**Table 6:** TBHQ quantification during extreme oxidation of FOBL (Op. TBHQ).

Oxidation Time, h	Antioxidant Content in FOBL Op. THBQ, mg·L^−1^
0	1732 ± 18
1	310 ± 36
3	144 ± 30
5	140 ± 27
8	161 ± 7

**Table 7:** The effect of the optimum TBHQ addition on quality parameters of biolubricant after oxidation (8 h).

Parameter	Control Sample	TBHQ Sample
Viscosity	+527%	+20%
Acid number	+720%	+21%
Absorbance at 230 nm	+400%	+19%
TBHQ content *	--	−93%

* Control samples did not have any TBHQ addition.

**Table 8:** Chemical conditions selected for biolubricant production.

Parameter	Units	Value
Reaction temperature	°C	160
Reaction time	min	120
Stirring rate	rpm	350
Catalyst concentration	%	1.0
FAME/PE mole ratio	n.a. *	3
Pressure	mmHg	260

* Not applicable.

## Data Availability

Not applicable.
